# Comparison of Polyphenol, Sugar, Organic Acid, Volatile Compounds, and Antioxidant Capacity of Commercially Grown Strawberry Cultivars in Turkey

**DOI:** 10.3390/plants10081654

**Published:** 2021-08-11

**Authors:** Ipek Urün, Sule Hilal Attar, Duygu Ayvaz Sönmez, Muhammet Ali Gündeşli, Sezai Ercişli, Nesibe Ebru Kafkas, Luna Maslov Bandić, Boris Duralija

**Affiliations:** 1 Department of Horticulture, Faculty of Agriculture, University of Çukurova, Balcali 01330, Turkey; ipek-016@hotmail.com (I.U.); shattar@cu.edu.tr (S.H.A.); ebru@cu.edu.tr (N.E.K.); 2 Yaltır Agricultural Products Inc., Sarıhuğlar 01355, Turkey; duygu.ayvaz@gmail.com; 3 Department of Plant and Animal Production, Nurdagı Vocational School, Gaziantep University, Gaziantep 27310, Turkey; maligun4646@gmail.com; 4 Department of Horticulture, Faculty of Agriculture, Ataturk University, Erzurum 25240, Turkey; sercisli@gmail.com; 5 Faculty of Agriculture, University of Zagreb, Svetosimunska 25, 10 000 Zagreb, Croatia; bduralija@agr.hr

**Keywords:** strawberry, sugars, organic acids, phenolic compounds, volatile compounds, SPME/GC/MS, HPLC

## Abstract

The aim of this study was to compare certain important fruit quality parameters such as sugars, organic acids, total phenolic content, antioxidant capacity, and volatile compounds of 10 commercial strawberry cultivars grown in the research and experimental area in Yaltir Agricultural Company located in Adana provinces of Turkey. As for the sugar content of strawberry fruits, fructose was identified as the dominant sugar and the highest value (4.43%) was found in the cultivar “Rubygem”. In terms of organic acid, among the examined strawberry cultivars, “Calinda” had the greatest level of citric acid (711.45 mg g^−1^). Regarding vitamin C content, the highest amount was found in the cultivar “Sabrina” (25.08 mg 100 g^−1^). Ellagic acid was the main phenolic acid in all examined cultivars (except the “Plared” cultivar), and the highest amount was detected in the “Fortuna” cultivar (3.18 mg 100 g^−1^). We found that the cultivar FL-127 had the highest total phenolic content (158.37 mg gallic acid equivalent 100 g^−1^ fresh weight base). Regarding antioxidant capacities, the highest value (88.92%) was found in the cultivar Victory among all the cultivars studied. The detailed analysis of volatile compounds was performed by gas chromatography/mass spectrometry (GC/MS) and 34 compounds were detected. Among them, esters, acids, and alcohols were found to be the major volatile compounds in strawberry fruits. In conclusion, strawberry fruits belong to ten cultivars showed abundant phenolic compounds and at the same time have high antioxidant activity.

## 1. Introduction

Over the past two decades, interest in research to determine health compounds in fruits has increased [1]. Colorful berries are rich in biochemical content and supply lots of health benefits for humans. They are rich in bioactive compounds such as phenolic acids, flavonoids, sugars, organic acids, and anthocyanins [2,3]. Strawberry is a member of the genus Fragaria (family: Rosaceae) and one of the most important economic berry fruits that are cultivated throughout the world. Today, it is widely cultivated, especially in Europe, and can be consumed not only fresh but also processed into marmalade, jam, fruit juice, and beverages. Strawberry fruits are an important source of health-promoting compounds for mankind. Among the berries, it is the most preferred due to its pleasant aroma, charming color, good taste, flavor, and bioactive compounds [4,5,6].

It is well known that phenolic composition strongly affects the quality of the fruits and contributes to both their sensory-organoleptic properties and their nutritional values. They affect the taste of products and create a sour taste. Anthocyanins are the most notable and quantitatively the most important type of polyphenol in strawberries. Anthocyanins is one of the phenolic compounds, are responsible for the bright red color of the fruit. The concentration and composition of anthocyanins are important for the sensory quality of fruits and products, in addition to their possible health benefits. Recent studies have focused on determining the bioactive compound of diverse strawberry varieties and reveal the best one and determining the factors that affect the composition of this unique fruit [7,8,9,10,11,12,13].

Carbohydrates (sugars) and organic acids are the major factors that influence the organoleptic properties of fruits. The organic acid–sugar ratio is also an important criterion to characterize fruit flavor. Organic acids show antioxidant properties, which explains their extensive use for pharmacological purposes. The organic acid content of strawberry fruits changes according to the genotypic background of the plant and the variety has a decisive role on the taste of the fruit by affecting the acid–sugar balance [14,15,16].

Fruit volatile compounds and taste are the results of a special assortment and mixture of different metabolites. While sugars and acids contribute to sweetness and tartness, the aroma is derived from combinations of volatile molecules. The different proportions of the volatile components and the presence or absence of trace components often determine aroma properties. The flavor of cultivated strawberries is mainly determined by a complex mixture of esters, aldehydes, alcohols, and sulfur compounds [17,18]. Because of their typical aroma, strawberries have always been the candidate of choice in aroma analysis. In addition, the chemical basis of strawberry flavor has been studied in depth. The flavor industry can supply synthetic flavors of many food types [18]. Aroma is one of the most essential ingredients in strawberries. However, recent research has shown that the contents of bioactive compounds depend largely on the strawberry variety, while seasonal changes have a comparative effect. In addition, these compounds show positive effects on human health due to antioxidant, antiallergic, anticarcinogenic, and antimicrobial effects of the fruit [11,12].

Turkey has become one of the world’s largest strawberry producers in recent years [19]. Strawberry varieties examined in the study are widely grown in Turkey and are valuable in European markets. These are the varieties commonly used in the juice processing industry and fresh market use. In addition, many local (native) varieties can be used as a source for breeding material with their special aroma and pleasant taste. In studies on strawberries in Turkey, their pomological and chemical properties were generally examined. Studies on organic acids, phenolic compounds, sugar, flavor, and antioxidant content of strawberry fruit are not sufficient. Therefore, the determination of phenolic compounds, organic acids, flavor, antioxidant capacities of these special varieties is an important research topic.

The study aimed to determine the carbohydrates (sugars), organic acids, total phenol content, individual phenolic compounds, total antioxidant capacity, and volatile aroma substances on ten strawberry varieties grown in Adana province of Turkey. These parameters are important in determining the quality of strawberries.

## 2. Materials and Methods

### 2.1. Plant Material

Rubygem, Fortuna, Festival, Calinda, FL 127, Plared, Sahara, Sabrina, Victory, and E-2 strawberry cultivars were used in the study. All cultivars are grown together at the experimental field of Yaltir Agricultural Company in Adana provinces of Turkey. About 30 fruits were homogeneously collected from each cultivar during the harvest period of May 2019. The samples were placed in cloth bags and then transferred to the laboratory for future analyses and stored at −80 °C in deep freeze. 

“Rubygem” is a short-day strawberry cultivar that was obtained from cross-breeding between cv. Earlibrite × cv. Carlsbad in Australia. It has an excellent sugar–acid balance gives it a sweet-tasting flavor. Attractive medium-sized generally conical to cordiform and short wedge-shaped fruits give good yields. The fruit flesh is firm, sweet, and flavorful. The fruit is glossy bright red on the outside and medium red on the inside. Resistance to Fusarium Wilt is displayed. 

“Fortuna”, originated from cross-breeding Winter Dawn × FL 99-35 by the University of Florida. Fortuna is moderately resistant to Colletotrichum crown rot and anthracnose fruit rots but is moderately susceptible to Botrytis fruit rot and is highly susceptible to Phytophthora root rot. It has large, firm, glossy fruit. 

The “Festival” is a cross between Rosa Linda × Oso Grande. The cultivar is distinguished by the numerous runners it produces in the fruiting field, the long pedicels attached to its fruit, and the production of fruit that are flavorful, firm-fleshed, deep red on the outside, bright red on the inside, conically shaped, and have large, showy calyces when grown in Dover, Florida or the other areas that have a subtropical climate similar to that of Dover.

“Calinda” is obtained in a planned breeding program conducted in The Netherlands and Bonares, Andalusia, Spain. Calinda is an early-season short-day cultivar that produces exceptionally attractive, flavors berries. 

“FL127” is cross between FL 05-107 and FL 02-58 in Florida, USA. FL 127 is distinctive for its combination of high early and total yields, large fruit size and excellent fruit quality due to high sugars, fruity aroma, firm but the juicy texture and extended shelf life. The plant is upright and moderately vigorous. This cultivar is resistant to anthracnose fruit rot and moderately resistant to charcoal rot and Colletotrichum crown rot but is susceptible to Botrytis fruit rot, powdery mildew, and Phytophthora root rot.

“E22” is distinct for its day-neutral flowering habit, low chill requirement and compact plant habit. Its fruits have excellent rain tolerance, very consistent shape and color, and very good flavor. It is resistant to Phytophthora crown rot but is susceptible to powdery mildew and anthracnose fruit and crown rots.

“Plared”, the variety of strawberry was created in a breeding program by crossing two parents in 2009 in Cartaya (Huelva), Spain between 2 unpatented progenies, “09-024” and “03-98”. It has a pleasant scent with a nice balance between sugar and acidity and it has an intense bright color and large fruit size. It is a short-day variety.

“Sahara” variety was created in a breeding program by crossing two parents in 2006 in (Huelva), Spain by 02.125 (unpatented) and 03.98 (unpatented). The Sahara fruits resemble the Sabrosa ones, but they are much earlier. It is a short-day variety.

“Sabrina” is obtained in a breeding program by crossing two parents, namely 9719 (unpatented) and 94-020 (unpatented). It has very firm and resistant skin, it enables long periods of conservation and transport. This variety produces red colored, conical-shaped, firm, large fruit.

“Victory” is an early variety with regular production throughout the whole season. It is a rustic variety that stands out mainly for the post-harvest life of its fruits, being able to reach the most distant destinations. The seedling cross was selected from a controlled breeding plot in Ventura County, California, the USA in the winter of 2004.

The production of certified strawberry varieties is carried out in the tissue culture laboratory by the meristem culture method. In another word, the production system is based on virus-free meristem seedlings. Solarization or fumigation is done during field experiments against soil-borne diseases. The experiment plantations included 2 years old strawberry plants. Licensed pesticides are used for strawberries throughout the production season. During the summer period, the control of the spider and aphid was done. After taking flowers and stems, fungicide is applied. In winter, spraying is done twice against Botrytis, the first at 25% of flowering and the second at the beginning of fruiting. Great attention has been paid to the red spider and thrips. Fresh and mature strawberry fruits were harvested at the ripe stage. A total of 30 fruits (10 fruits per replicate) from each variety were selected, with uniform size, shape, color without any visible damage, a disease for further study. The experimental area’s soil is processed deeply (at least 50–60 cm) and the plow layer (hard layer) is broken. Disinfection is done. The soil is aerated, plowed with a cultivator and the clods are broken. Very well burned animal manure was given 3–4 tons per decare (before soil disinfection). Base fertilizer is given as per da: 4 kg Nitrogen, 7 kg Phosphorus and 20 kg of Potassium. Fertilization begins 3 weeks after planting. 0.20–0.25 kg/da Nitrogen is given per decare per day. This practice continues until November. No water or fertilizer is given between November and January. Starting from February, 0.20–0.25 kg/da of Nitrogen and 0.25–0.40 kg/da of Potassium are given daily depending on the soil temperature. As soon as chlorosis (yellowness) is seen from planting, 500 gr. Sequestrene Fe (Iron) or others are given by drip irrigation and this process is repeated as needed. 

### 2.2. Biochemical Parameters

#### 2.2.1. Determination of Total Polyphenolics

The total phenolic content was determined by the Folin-Ciocalteau method [20] with some modifications. The analysis was done with a UV/VIS spectrophotometer (Thermo Fisher Scientific, FI-01620 Vantaa, Finland). Briefly, 9 mL of 80% methanol was added to 1 mL of the juice sample. The mixture was centrifuged at 5500 rpm for 10 min. A volume of 50 µL of supernatant was added to 250 µL of Folin-Ciocalteu reagent. Afterward, 750 µL 20% (*w*/*v*) Na_2_CO_3_ were supplemented, and it was incubated 2 h at room temperature. Then, the absorbance was measured at 760 nm against a blank. Quantifications were calculated through a calibration curve daily prepared with known concentrations of gallic acid (GA) standards, and results are expressed as mg GAE 100 g^−^^1^ fresh weight (FW) of strawberry.

#### 2.2.2. Determination of Total Antioxidant Capacity

The radical scavenging activity of DPPH (2,2-Diphenyl-1-picrylhydrazyl) was done as described by [10] with slight modifications. Briefly, 0.06 µM of ethanolic DPPH was freshly prepared. Then, 1950 µL of DPPH• was added to 50 µL of strawberry juice sample. The mixture was shaken for 1 min and kept in the dark for 30 min at room temperature. Absorbance was measured against the blank reagent at 515 nm. Radical scavenging activity %DPPH inhibition was calculated using the following equation:

%Inhibition = 100 × [(Abs blank (t = 30)) − (Abs sample)]/[(Abs blank (t = 30))](1)

where Abssample was the absorbance of the reaction in presence of sample (sample dilution + DPPH solution), Absblank was the absorbance of the blank for each sample dilution (sample dilution + DPPH solvent), and Abscontrol was the absorbance of control reaction (sample solvent + DPPH solution).

#### 2.2.3. Determination of Carbohydrate (Sugar) Content 

Glucose, fructose, xylose, sucrose, and total sugar content in the juice obtained from the harvested strawberries were determined according to the method developed by Crisosto et al. [21]. Before analysis, frozen juice samples were thawed at 25 °C. A volume of 1 mL of juice was added to 4 mL of ultrapure water (Millipore Corp., Bedford, MA, USA). The reaction mixture was placed in an ultrasonic bath and sonicated at 80 °C for 15 min and then centrifuged at 5500 rpm for 15 min and it was filtered before HPLC analysis (Whatman nylon syringe filters, 0.45 µm, 13 mm, diameter). The high-performance liquid chromatographic apparatus (Shimadzu LC 20A VP, Kyoto, Japan) consisted of an in-line degasser, pump, and controller coupled to a Refractive index detector (Shimadzu RID 20A VP, Kyoto, Japan) equipped with an automatic injector (20 µL injection volume) interfaced to a PC running Class VP chromatography manager software (Shimadzu, Kyoto, Japan). Separations were performed on a 300 mm × 7.8 mm i.d., 5 µm, reverse-phase Ultrasphere Coregel-87 C analytical column (Transgenomic) operating at 70 °C with a flow rate of 0.6 mL min^−1^. Elution was isocratic with ultrapure water. Individual sugars were calculated based on their standards and expressed in % of fresh weight (FW).

#### 2.2.4. Determination of Organic Acids

Organic acids in strawberry fruit juice were determined by the HPLC analysis by Bozan et al. [22]. The malic, citric, succinic, fumaric, and L-ascorbic acid contents in strawberry juice samples were determined. For organic acids extraction, 1 mL of the sample, and 4 mL of 3% metaphosphoric acid were mixed. The mixture was placed in the ultrasonic water bath at 80 °C for 15 min and it was sonicated and centrifuged at 5500 rpm for 15 min. Afterward, the mixture was filtered (Whatman nylon syringe filters, 0.45 µm, 13 mm, diameter) prior to HPLC analysis. The extract of organic acids was analyzed using a high-performance liquid chromatographic apparatus HPLC (Shimadzu LC 20A VP, Kyoto, Japan) equipped with a UV detector (Shimadzu SPD 20A VP, Kyoto, Japan) and we used an 87 H column (5 μm, 300 mm × 7.8 mm (I.D.), Transgenomic). As for the operating conditions column temperature, was set at 40 °C; injection volume, 20 μL; detection wavelength, 210 nm; flow rate 0.8 mL/min. and 0.05 mM sulphuric acid was used as the solvent. Identification of organic acids and determination of peaks is based on the retention times of peaks and comparison of spectral data according to standards. The identified acids were evaluated according to the relevant standard calibration curves. Results are expressed as mg 100 g^−^^1^.

#### 2.2.5. Determination of Phenolic Compounds

For the extraction and hydrolysis procedure of phenolics of homogenized samples, boiling refluxed was used for 1 h [23]. After it was cooled, the mixture was filtered and made up to 10 mL with 20% acetone. These samples were directly used for HPLC analyses. The liquid chromatographic apparatus (HPLC, Shımadzu LC-20A, Kyoto, Japan) consisted of an in-line degasser, pump, and controller coupled to a UV detector equipped with an automatic injector (20 μL injection volume) interfaced to a PC running ChemStation chromatography manager software. Separations were performed on a 150 mm × 4.6 mm i.d., 5 μm, reverse-phase Nucleosil C18 analytical column (Supelco, PA) operating at room temperature with a flow rate of 1 mL/min. Detection was carried out with a sensitivity of 0.1 a.u.f.s. between the wavelengths of 280 and 360 nm. Elution was using a nonlinear gradient of the solvent mixture 2.5% HCOOH in water (solvent A) and 2.5% HCOOH in acetonitrile (solvent B). All of the samples were directly injected into the reverse phase chromatography column after filtration. Gallic acid, myricetin, caffeic acid, *p*-coumaric acid, ellagic acid, quercetin, and kaempferol were dissolved in methanol at a concentration of 1 mg mL^−^^1^, and five dilute solutions from these stock solutions were used to prepare calibration curves of each standard. Three replicates from each sample were used for HPLC analyses. All samples and standards were injected three times, and mean values were used.

#### 2.2.6. Determination of Volatile Compounds 

Volatile compounds were extracted by solid-phase microextraction (SPME). One gram of homogenate strawberry was weighed and 1 mL of CaCl_2_ was added in headspace vial for 30 min at 40 °C. The SPME fiber 85 μm CAR/PDMS (Carboxen/Polydimethylsiloxane; light blue) was used for analysis. The adsorbed flavor compounds of the strawberry fruits were analyzed using a Shımadzu GC-2010 Plus Gas chromatography-mass spectrometer (GC/MS). HP-Innowax Agilent column (30 m × 0.25 mm i.d., 0.25 µm thickness) was used and helium was the carrier gas. The GC oven temperature was kept at 40 °C and programmed to 260 °C at a rate of 5 °C/min, and then kept constant at 260 °C for 40 min. The injector temperature was at 250 °C. The MS was taken at 70 eV. The mass range was *m*/*z* 30–400. A library search was carried out using the commercial Wiley, NIST, and Flavor GC–MS Libraries. The mass spectra were also compared with those of reference compounds and confirmed with the aid of retention indices from published sources. Relative percentage amounts of the separated compounds were calculated from total ion chromatograms. 

### 2.3. Statistical Analysis

All the result data were processed with the SPSS package program version 16.0 (SPSS Inc., Chicago, IL, USA). All data were presented as the mean ± standard error (SE) and analyzed by one-way analysis of variance (ANOVA). Differences were considered significant at *p* < 0.05. Principal component analysis (PCA) was applied to examine the relationship between phenolic compounds data and strawberry samples. Mathematica program for Windows version 4.0 was used for data processing (Wolfram Research, Champaign, IL, USA).

## 3. Results and Discussion

There were statistically significant differences among the strawberry varieties in terms of studied all biochemical traits (Table 1, Table 2, Table 3 and Table 4). 

### 3.1. Total Phenolic Content and Antioxidant Capacity 

The results of total phenolic of 10 strawberry cultivars are given in Table 1. Statistically significant differences were found among varieties at *p* < 0.05. The total phenolic content of strawberry varieties varied from 99.02 to 158.37 mg GAE 100 g^−^^1^ FW (Table 1). FL-127 had the highest total phenolic content (158.37 mg GAE 100 g^−^^1^ FW), while Plared had the lowest (99.02 mg GAE 100 g^−^^1^ FW). The previous study [24] showed that strawberry fruits have a good source of total phenolics, and values agreed with our results. 

There were significant differences in terms of antioxidant capacities of examined varieties grown in the Adana region in the present study (*p* < 0.05). The findings indicated that the Victory and Rubygem varieties had the highest antioxidant capacity with 88.92% and 88.58% among the studied varieties, respectively. Researchers have demonstrated that a high antioxidant activity correlated with high phenolic content [24,25,26]. In a study [27] two commercial strawberry cultivars namely, Rubygem and Osmanlı, which grows in Osmaniye in southern Turkey, used and results showed that the total phenolic content in fruits of varieties were in range of 305 and 377 mg GAE 100 g^−^^1^, the total flavonoid amount 121.7 and 36.5 mg kg^−^^1^, respectively. They reported 91.0% and 58.6% in terms of DPPH scavenging activity among varieties. Our results in accordance with [27]. Strawberries were also previously reported that higher in antioxidant capacity than the other berry species, such as gooseberry and raspberry [28].

### 3.2. Carbohydrate (Sugars) and Total Soluble Solid Content 

Carbohydrate of strawberries are the main taste compounds and are considered as one of the major fruit quality parameters preferred by both producers and customers. In the present study, the values of carbohydrates (sucrose, glucose, xylose, fructose, total sugar) in fruits of ten strawberries were measured. Data related to sugars, total sugar, and °Brix are shown in Table 2. There were statistically significant differences among varieties in terms of all specific sugars, total sugars except °Brix (*p* < 0.05).

In the fruits of ten strawberry varieties, glucose and fructose are the major sugars with values ranging from 1.69 ± 0.01 to 3.93 ± 0.13 g 100 g^−1^ FW and 2.17 ± 0.13 to 4.43 ± 0.21 g 100 g^−1^ FW, respectively. While the content of sucrose and xylose is relatively much lower with the values ranging from 0.36 ± 0.11 to 1.94 ± 0.10 g 100 g^−1^ FW and 0.01 ± 0.00 to 0.16 ± 0.19 g 100 g^−1^ FW, respectively. Here, we found the highest values of both glucose and fructose in “Rubygem”, while the lowest content of glucose and fructose were both detected in “Plared” variety. The majority of the rest varieties like “Calinda”, “Sahara”, “Sabrina”, “Victory” and “E-22” showed similar glucose and fructose content varied from 2.60 to 3.10 g 100 g^−1^ FW, respectively. The results also indicate the content of glucose and fructose in strawberries are closely related to each other and varieties with high glucose contents also showed high fructose values. For sucrose, the values ranged from 0.36 ± 0.11 to 1.94 ± 0.10 g 100 g^−1^ FW, and the lowest and the highest sucrose content value was obtained in “Festival” and “Victory”, respectively. Xylose content is relatively lower compared to fructose, glucose, and sucrose. The highest xylose content was in E-22 with a value of 0.16 ± 0.19 g 100 g^−1^ FW and the lowest was in FL-127 with the value of 0.01 ± 0.00 g 100 g^−1^ FW. Actually, except “Calinda” and “E-22”, xylose content in the rest of the varieties was similar and quite low. Literature studies showed some differences and little consistency in sugar concentrations compared to our study [29,30]. Previously fructose (2.55–2.86 g 100 g^−1^ FW) was found to be the predominant sugar in strawberry fruit of two cultivars “Korona” and “Tufts” followed by glucose (1.79–2.25 g 100 g^−1^ FW) and sucrose (0.01–0.25 g 100 g^−1^ FW), respectively [29] indicated similarities with our study. In another study, the predominant sugar was found as glucose (1.6–1.8 g 100 g^−1^ FW) in two strawberry varieties (Festival and Ventana) and followed by sucrose (0.9–1.1 g 100 g^−1^ FW) and fructose (0.9–1.2 g 100 g^−1^ FW) [30]. Camaraso and Selva varieties had fructose and glucose as 3.1–5.5 g 100 g^−1^ FW and 3.5–6.7 g 100 g^−1^ FW and sucrose 0.6–0.9 g 100 g^−1^ FW, respectively [31]. The composition of soluble sugars in 10 strawberries in the present study showed a similar result compared to previous studies [32,33]. The total sugar content (including sucrose, glucose, fructose, and xylose) was high in variety “Rubygem” (8.78 ± 0.37 g 100 g^−1^) and “FL-237” (8.46 ± 1.02 g 100 g^−1^). Varieties “Festival”, “Calinda”, “Sabrina”, “Victory”, and “E-22” presented medium values from 7.25 ± 0.33 g 100 g^−1^ to 7.86 ± 0.35 g 100 g^−1^, while varieties like “Fortuna”, “Plared”, and “Sahara” attained relatively low total sugar content (from 5.13 ± 0.15 to 6.70 ± 0.51 g 100 g^−1^ FW). Mahmood et al. [29] reported the total sugar contents between 3.61% and 5.36% in two strawberry cultivars. The soluble solid (°Brix) ratio of 10 varieties was analyzed and four varieties (“Rubygem”, “Calinda”, “Sabrina” and “Victory”) showed the highest value (10.50% ± 0.71%), “Festival”, “FL-127”, “Sahara” and “E-22” showed a similar °Brix value (from 9.00% ± 1.41% to 9.50% ± 0.71%), while variety “Plared” and “Fortuna” had low soluble solid contents (°Brix values were 8.00% and 6.00%, respectively). According to [29] the strawberry fruits had 4.31–10.11% for °Brix values that show close values with our study. In another study, [24] reported that the soluble solids content of four strawberries was variable and ranged from 5.50 to 7.17 °Brix. 

### 3.3. Organic Acids

Organic acids are known to affect the formation of taste and many physiological processes in fruits that depends on the variety. The sugar–acid balance and contents are very important in determining the taste characteristics of the fruit. Oxalic acid, L-ascorbic, citric, malic, fumaric, and succinic acid contents in fruits of 10 strawberry cultivars were examined in this study and results are given in Table 3. There were statistically significant differences among varieties in terms of organic acid contents (*p* < 0.05). According to the organic acid results, citric acid is the major and predominant organic acid in fruits of 10 strawberry varieties, which ranges from 522.40 ± 1.5 to 711.45 ± 2.11 mg 100 g^−1^ FW. The highest citric acid content was obtained in “Calinda” and the lowest in “FL 127”. L-ascorbic acid was detected at concentration 13.90 ± 0.641 to 25.08 ± 0.35 mg 100 g^−1^ FW, malic acid ranges from 159.80 ± 2.84 to 266.65 ± 0.30 mg 100 g^−1^ FW, and succinic acid was from 24.27 ± 1.81 to 59.53 ± 2.92 mg 100 g^−1^ FW, respectively. Fumaric acid was also detected and presented in very small amounts (from 1.48 ± 0.05 to 2.10 ± 0.06 mg 100 g^−1^ FW), therefore it might not significantly influence the fruit flavor of strawberry. Several previous studies showed similar results with our present data and revealed that citric acid is widely found as the principal organic acid in strawberries [12,34,35,36]. Malic acid is also a major member during strawberry fruit development and the concentration of malic acid is much higher at the green or developing stage than ripen stage. However, several previous results showed the content of malic acid at the ripe stage is much higher than our result [12,37]. Only Mahmood et al. [29] reported a similar result with our data, and this might be caused the co-elution with fructose during the determination of malic acid [32]. Succinic acid is also very abundant in strawberries. Moreover, [38] compared the succinic acid in six strawberries, and values ranged from 0.12–0.25 mg 100 g^−1^ FW, however, this result is much lower than our study. L-Ascorbic acid has important antioxidant and metabolic functions [12]. In agreement with our results, fumaric acid has been reported as the least abundant organic acid in strawberries [32]. The findings of this study are mostly in agreement with those of other researchers indicating the organic acid richness of berry fruits [38,39,40,41]. The minor differences are possibly attributed to environmental conditions and genetic factors of the studied species and varieties.

### 3.4. Phenolic Compounds

Phenolic compounds are plant metabolites that spread throughout the plant kingdom. The recent focus on phenolic compounds stems from their potential protective role against oxidative damage diseases such as coronary heart disease, stroke, and cancer, through the ingestion of fruits [42]. Phenolic compounds are secondary plant metabolites with strong antioxidant activities that widely exist in fruits and vegetables, especially in berries [1,43]. Particularly in dense black-purple and red-colored fruits such as blueberries, black currants, blackberries, strawberries, and raspberries are a rich source of phenolic compounds and antioxidants [44].

In this study, phenolic compound in strawberry fruits was identified and quantified by HPLC. Seven phenolic compounds were determined in the fruits of strawberry varieties. Statistically significant differences were recorded among the phenolic contents of the varieties. Among these, ellagic acid contents ranged between 0.49 and 3.18 mg 100 g^−1^ FW, gallic acid contents between 0.45 and 1.06 mg 100 g^−1^ FW, quercetin contents between 0.84 and 1.15 mg 100 g^−1^ FW, myricetin contents between 0.19 and 1.94 mg 100 g^−1^ FW, caffeic acid contents between 0.13 and 0.23 mg 100 g^−1^ FW, *p*-coumaric acid contents between 0.05 and 0.35 mg 100 g^−1^ FW, and kaempferol contents between 0.04 and 0.18 mg 100 g^−1^ FW, respectively (Table 4).

Ellagic acid was the predominant phenolic compound in Fortuna, Calinda, and Victory varieties. Gallic acid was the predominant phenolic compound in Fortuna, Victory, and E-22 varieties. Caffeic acid was the predominant phenolic compound in Festival, Victory, and FL-127 varieties. Quercetin was the predominant phenolic compound in Fortuna, Sahara, and FL-127 varieties. It is assumed that ellagic acid has antioxidant, anti-mutagenic, anti-inflammatory, and cardioprotective activities [45]. According to our results, ellagic acid was the major phenolic compound in fruits of ten strawberry varieties and significant differences were determined among the strawberry varieties concerning phenolic compound distribution. These differences are likely to be due to variety-specific characteristics because all varieties found the same conditions. Similar results have been reported in many other strawberry varieties [46,47,48]. Ref [7] evaluated ellagic acid content in several strawberry varieties which ranged from 28.6–68.4 mg kg^−1^ FW, also [48] reported the ellagic acid contents varied between 0.61 ± 0.05 and 2.60 ± 0.05 mg 100 g^−1^ FW. Ref [49] reported that the gallic acid, procatechinic, vanillic acid, caffeic acid, *o*-coumaric acid, and *p*-coumaric acid, and ferulic acids in black mulberry fruits were as 27.3 mg kg^−1^ FW, 121.8 mg kg^−1^ FW, 6.5 mg kg^−1^ FW, 117.2 mg kg^−1^ FW, 212.7 mg kg^−1^ FW, 761.8 mg kg^−1^ FW, and 34.1 mg kg^−1^ FW, respectively, indicating richness of berries in terms of phenolic compounds. 

The PCA was generated to correlate phenolic content and antioxidant capacity with ten cultivars of strawberry. Two main principal components (PCs) were selected giving 54% of total variability. 

**Figure 1:**
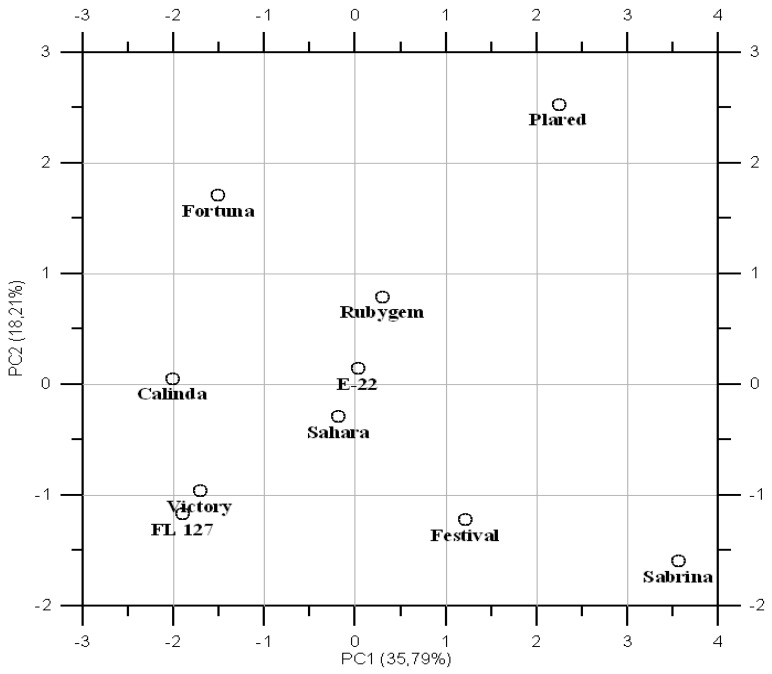
Principal component analysis (PCA) for different varieties and phenolic profile (individual phenolic compounds, total phenols content, and DPPH).

As indicated in Figure 1, Victory and FL127 were located close to each other. Sahara was located closer to E-22 which indicates similarities among these cultivars in terms of a specific chemotaxonomic pattern of phenolic compounds. The rest of the analyzed cultivars were situated in different parts of the plot, which reflects differences between their characteristics. It can be concluded that the relationship between the compounds present in the fruit is variety-specific.

Differences among fruits for the quantity and the composition of phenolic compounds can be due to factors as genotype, environmental conditions, agro techniques, and storage conditions as observed by [50,51,52,53] in berry fruits.

### 3.5. Volatile Compounds

In the present study, a total of 34 volatile compounds were observed in 10 strawberry varieties by using different HS/SPME/GC-MS techniques and the results were given in Table 5. Totally 6 esters, 3 aldehydes, 1 terpene, 9 acids, 6 alcohols, 6 ketones, and 3 other compounds were identified in the current study. According to the results, esters, acids, and ketone were found to be the major volatiles in strawberry fruits. As seen in Table 5, the percentage of the volatile compounds ranged from 3.27% to 31.99% for alcohols, from 1.05% to 21.37% for esters, from 9.35% to 68.45% for ketones, from 0.34% to 2.61% for terpenes, for 20.20% to 52.43% for acids, and from 1.05% to 21.37% for esters. As shown in Table 5, the amounts of esters, aldehydes, acids, alcohols, terpenes, ketones from aroma compounds varied among varieties. Compounds 2-ethyl-1-hexanol, terpineol, 1-dodecanol, nerolidol, linalool, and geraniol were detected as alcohol. Nerolidol was found to have the highest proportion (Table 5). In different fruits, it has been determined that alcohols contribute a little to aromas [54]. Gamma decalactone was detected in high amount as ketone. 

The varieties were also rich in butanoic acid and hexanoic acid, which are important volatile substances in strawberries. Hexanoic acid was the predominant acid in 10 varieties, followed by acetic acid and butanoic acid. Among the ten varieties, Calinda had the highest concentration of aldehydes including 2,4-dimethyl-benzaldehyde. Ester compounds, including diethyl ester, ethyl ester, and methyl ester are important aroma compounds. The esters detected in the ten strawberry varieties were diethyl ester phenylmethyl ester acetate, octyl ester ethyl ester, and methyl ester. In this study, especially esters such as diethyl ester and methyl ester were the most abundant and high percentage of esters. Esters provide fruity and floral characters in fruits and the amount of these compounds provides an important basis for classification [27,55]. Similar results were previously reported by [12] for aroma compounds in strawberries. Previous studies have also shown that strawberry fruits have many free volatile compounds. The number of volatiles is on the same level as those previously extracted from strawberries with the dynamic headspace technique. The typical aroma of strawberries does not come from just one or a few potent flavoring compounds but from a large number of volatiles in specific concentrations and a certain balance between them. The strawberry flavor is therefore the result of the combined perception of many aromatic components [56]. Although over 360 compounds have been identified in strawberry flavor, only a few volatile substances (primarily methyl and ethyl esters) appear to be the most important contributors to the strawberry flavor [27,56,57,58,59,60].

## 4. Conclusions

In this study, bioactive and aroma properties of different commonly grown strawberry varieties in Adana province in Mediterranean were examined and nutritional values and importance of these varieties for human health were determined using advanced analysis techniques. The region of study is one of Turkey’s most important regions in terms of citrus production. Demand for fruit types containing more antioxidants is increasing due to the identification of biochemical compounds that have an important impact on human health in recent studies. This situation increases the importance of strawberries in terms of human health. In this study, 10 different varieties were evaluated under the same climatic and cultural conditions and provide a valuable reference for further studies how genotype affects bioactive and aroma composition in strawberries. Studies on the biochemical properties of strawberry cultivars are important to find the most suitable for commercial production and human health. According to the results, it was determined that Rubygem, Victoria, and FL-127 varieties stand out in terms of bioactive properties including phenolic profiles, the antioxidant capacity and have higher sugar composition and acidity (%) values. The results show high potential of strawberry fruits for health benefits. Finally, differences found among varieties highlight a great variability and should be considered for the choice of the variety and in breeding programs aimed at selecting varieties with improved antioxidant capacity and nutraceutical properties.

## Figures and Tables

**Table 1:** Total phenol (mg GAE 100 g^−1^ FW) and antioxidant activity (% DPPH inhibition) in fruits of ten strawberry varieties.

	Rubygem	Fortuna	Festival	Calinda	FL-127	Plared	Sahara	Sabrina	Victory	E-22	LSD_0.05_
**TP**	105.46 ± 1.48 g	122.66 ± 1.55 f	141.86 ± 1.48 bc	156.65 ± 1.49 a	158.37 ± 2.70 a	99.02 ± 2.33 h	139.42 ± 0.49 cd	136.77 ± 1.90 d	142.63 ± 1.56 b	129.24 ± 1.95 e	3.04 **
**%DPPH**	88.58 ± 1.01 a	86.75 ± 0.71 cd	86.83 ± 0.50 bcd	88.21 ± 0.98 ab	88.59 ± 0.76 a	86.05 ± 1.74 d	86.20 ± 0.08 d	86.46 ± 0.47 cd	88.92 ± 0.42 a	87.65 ± 0.69 abc	1.45 *

Each value is expressed as mean ± standard deviation. TP: total phenol; % DPPH: DPPH inhibition. Different letters in the same row shows significant (*p* < 0.01) differences. Asterisks (*, **) indicate significant differences by LSD test.

**Table 2:** The sugars (%), total sugar (%) and °Brix in fruits of ten strawberry varieties.

	Rubygem	Fortuna	Festival	Calinda	FL-127	Plared	Sahara	Sabrina	Victory	E-22	LSD_0.05_
**Sucrose**	0.40 ± 0.03 g	0.74 ± 0.10 e	0.36 ± 0.11 g	1.48 ± 0.03 d	0.59 ± 0.03 f	0.82 ± 0.15 e	0.79 ± 0.10 e	1.64 ± 0.18 c	1.94 ± 0.10 a	1.77 ± 0.01 b	0.12 **
**Glucose**	3.93 ± 0.13 a	1.93 ± 0.02 f	3.15 ± 0.21 c	2.84 ± 0.12 d	3.57 ± 0.35 b	1.69 ± 0.01 g	2.69 ± 0.17 de	2.68 ± 0.14 de	2.52 ± 0.26 e	2.63 ± 0.04 de	0.21 **
**Xylose**	0.03 ± 0.00 b	0.03 ± 0.00 b	0.03 ± 0.00 b	0.14 ± 0.15 a	0.01 ± 0.00 b	0.02 ± 0.00 b	0.04 ± 0.02 b	0.03 ± 0.01 b	0.02 ± 0.00 b	0.16 ± 0.19 a	0.93 *
**Fructose**	4.43 ± 0.21 a	2.42 ± 0.03 f	3.71 ± 0.22 b	3.40 ± 0.12 bc	4.28 ± 0.63 a	2.17 ± 0.13 f	3.19 ± 0.22 cde	3.30 ± 0.10 cd	3.02 ± 0.28 de	2.93 ± 0.29 e	0.32 **
**T.sugar**	8.78 ± 0.37 a	5.13 ± 0.1 e5	7.25 ± 0.33 cd	7.86 ± 0.35 bc	8.46 ± 1.02 ab	5.71 ± 1.37 e	6.70 ± 0.51 d	7.65 ± 0.43 c	7.50 ± 0.43 c	7.50 ± 0.13 c	0.75 **
**°Brix**	10.50 ± 0.71	6.00 ± 0.00	9.50 ± 0.71	10.50 ± 0.71	9.50 ± 0.71	8.00 ± 0.00	9.50 ± 0.71	10.50 ± 0.71	10.50 ± 0.71	9.00 ± 1.41	

Each value is expressed as mean ± standard deviation. Different letters in the same row show significant (*p* < 0.01) differences. Asterisks (*, **) indicate significant differences by LSD test.

**Table 3:** The content of organic acids in fruits of ten strawberry varieties (mg per 100 g^−^^1^ FW).

	Strawberry Varieties										
	Rubygem	Fortuna	Festival	Calinda	FL 127	Plared	Sahara	Sabrina	Victory	E-22	LSD_0.05_
**A.A**	21.02 ± 0.42 cd	13.90 ± 0.641 f	19.59 ± 1.48 e	20.72 ± 0.55 de	22.16 ± 0.55 bc	15.035 ± 0.450 f	21.582 ± 0.43 cd	25.08 ± 0.35 a	21.31 ± 1.20 cd	23.053 ± 0.55 b	1.29 **
**Citric**	556.4 ± 04.17 d	536.65 ± 23.84 d	667.75 ± 53.66 b	711.45 ± 10.55 a	522.4 ± 7.50 d	670.4 ± 7.49 b	627.7 ± 14.46 c	648.05 ± 4.04 bc	636.15 ± 10.71 bc	535.25 ± 1.92 d	34.23 **
**Malic**	206.95 ± 2.21 cd	159.8 ± 14.22 f	236.95 ± 19.47 b	219.4 ± 5.37 c	242.6 ± 2.14 b	186.25 ± 4.59 e	202.55 ± 4.89 d	250.65 ± 1.72 b	238.75 ± 7.35 b	266.65 ± 1.50 a	14.48 **
**Succinic**	39.28 ± 2.58 ef	28.49 ± 2.50 gh	59.53 ± 2.92 a	36.55 ± 2.57 f	24.27 ± 1.81 h	30.14 ± 0.85 g	41.13 ± 3.55 de	43.73 ± 2.38 d	50.383 ± 0.44 c	55.05 ± 2.17 b	4.40 **
**Fumaric**	1.66 ± 0.08 bcd	1.52 ± 0.12 cd	1.48 ± 0.28 d	1.82 ± 0.08 b	1.48 ± 0.05 d	1.49 ± 0.11 d	1.66 ± 0.08 bcd	2.03 ± 0.03 a	1.71 ± 0.09 bc	2.10 ± 0.06	0.21 **

Each value is expressed as mean ± standard deviation. Different letters in the same row show significant (*p* < 0.01) differences. A.A: L-Ascorbic acid. Asterisks (**) indicate significant differences by LSD test.

**Table 4:** The content of phenolic compounds (mg 100 g^−1^ FW) in fruits of ten strawberry varieties.

Phenolic	Strawberry Varieties										
	Rubygem	Fortuna	Festival	Calinda	FL-127	Plared	Sahara	Sabrina	Victory	E-22	LSD_0.05_
**Gallic Acid**	0.65 ± 0.15 ef	0.97 ± 0.11 ab	0.79 ± 0.06 cde	0.56 ± 0.05 fg	0.45 ± 0.05 g	0.55 ± 0.10 fg	0.85 ± 0.04 bcd	0.73 ± 0.11 de	0.93 ± 0.10 abc	1.06 ± 0.02 a	0.14 **
**Caffeic Acid**	0.13 ± 0.05 b	0.16 ± 0.02 ab	0.23 ± 0.05 a	0.15 ± 0.05 b	0.20 ± 0.03 ab	0.15 ± 0.10 b	0.19 ± 0.0 b	0.17 ± 0.04 ab	0.19 ± 0.02 ab	0.16 ± 0.01 ab	0.07
***p*-coumaric Acid**	0.20 ± 0.01 ab	0.25 ± 0.02 ab	0.17 ± 0.01 ab	0.15 ± 0.02 b	0.10 ± 0.01 b	0.35 ± 0.22 ab	0.07 ± 0.04 b	0.06 ± 0.01 a	0.05 ± 0.04 b	0.16 ± 0.14 ab	1.50
**Ellagic Acid**	1.44 ± 0.93 d	3.18 ± 0.03 a	0.49 ± 0.04 e	2.55 ± 0.08 ab	2.22 ± 0.14 bc	0.61 ± 0.11 e	1.68 ± 0.20 cd	0.59 ± 0.07 e	2.52 ± 0.68 b	1.55 ± 0.0 d7	0.63 **
**Myricetin**	0.47 ± 0.14 cde	1.94 ± 0.08 a	0.19 ± 0.07 e	1.81 ± 0.16 ab	0.85 ± 0.18 c	0.81 ± 0.13 cd	0.32 ± 0.16 e	0.46 ± 0.03 de	1.49 ± 0.02 b	0.45 ± 0.62 de	0.37 **
**Quercetin**	0.84 ± 0.09 c	1.15 ± 0.12 a	0.91 ± 0.04 abc	1.03 ± 0.15 abc	1.11 ± 0.34 ab	0.97 ± 0.08 abc	1.07 ± 0.08 abc	0.88 ± 0.19 bc	0.91 ± 0.08 abc	0.95 ± 0.12 abc	0.25
**Kaempferol**	0.06 ± 0.01 c	0.09 ± 0.05 bc	0.11 ± 0.07 bc	0.07 ± 0.01 bc	0.04 ± 0.00 c	0.05 ± 0.01 c	0.08 ± 0.02 bc	0.11 ± 0.02 a	0.18 ± 0.02 b	0.08 ± 0.01 bc	0.10 **

Each value is expressed as mean ± standard deviation. Different letters in the same row shows significant (*p* < 0.01) differences. Asterisks (**) indicate significant differences by LSD test.

**Table 5:** The volatile compounds (μg kg^−1^) in fruits of ten strawberry cultivars.

Volatile Compounds	Strawberry Cultivars									
	Rubygem	Fortuna	Festival	Calinda	FL 127	Plared	Sahara	Sabrina	Victory	E-22
**Alcohols**										
2-ethyl-1-hexanol.	n.d	0.28	0.58	0.81	n.d	n.d	n.d	n.d	n.d	n.d
alpha-terpineol	1.88	1.75	0.55	4.94	4.08	1.79	0.67	0.88	0.50	n.d
1-dodecanol	0.27	n.d	0.46	2.23	1.17	n.d	0.19	0.30	n.d	n.d
Nerolidol	9.33	10.67	26.66	10.88	4.39	5.31	4.43	5.58	2.77	3.44
L-linalool	6.55	2.39	2.90	1.50	2.97	1.36	0.76	1.90	n.d	0.85
Geraniol	0.38	0.77	0.84	n.d	0.31	1.77	n.d	0.53	n.d	0.68
**Total Alcohols**	**18.41**	**15.86**	**31.99**	**20.36**	**12.92**	**10.23**	**6.05**	**9.19**	**3.27**	**4.97**
**Ketons**										
Gamma-dodecalactone	n.d	1.75	5.55	1.14	n.d	n.d	3.36	2.23	1.74	5.27
Gamma-decalactone	24.48	n.d	10.02	n.d	26.11	n.d	62.76	42.38	8.49	39.18
Dihydro-carvone	6.94	7.29	0.55	0.59	5.13	2.05	1.37	2.31	1.56	n.d
Menthalactone	n.d	0.20	0.76	0.69	0.58	n.d	0.16	0.36	0.47	0.72
diphenyl-methanone	0.59	1.22	1.30	0.73	1.21	2.88	0.80	0.88	1.27	n.d
5-hexyldihydro-2(3H)-furanone	0.44	7.35	2.28	6.20	n.d	9.12	n.d	n.d	9.55	n.d
**Total Ketons**	**32.45**	**17.81**	**20.46**	**9.35**	**33.03**	**14.05**	**68.45**	**48.16**	**23.08**	**45.17**
**Esters**										
1.2-Benzenedicarboxylic acid diethyl ester	2.68	1.05	10.20	6.60	13.66	7.93	0.8	7.72	7.76	0.82
Acetic acid phenylmethyl ester	n.d	n.d	n.d	0.94	1.06	2.86	0.52	0.35	0.47	0.55
2-Hexen-1-ol acetate.	0.44	n.d	n.d	0.91	0.71	n.d	n.d	n.d	1.29	0.55
Butanoic acid octyl ester	n.d	n.d	1.12	n.d	0.76	n.d	0.53	n.d	n.d	1.17
Hexanoic acid ethyl ester	5.01	n.d	0.99	0.93	2.42	9.98	0.49	0.82	0.60	1.64
Hexanoic acid methyl ester	6.45	n.d	1.75	5.86	2.76	n.d	0.68	1.62	1.52	1.63
**Total Esters**	**14.58**	**1.05**	**14.06**	**15.24**	**21.37**	**20.77**	**3.01**	**10.51**	**11.64**	**6.36**
**Acids**										
2-methyl-butanoic acid	0.60	2.95	2.92	n.d	1.14	1.65	0.63	2.08	4.29	0.95
Acetic acid	6.81	8.73	6.76	7.16	3.49	19.59	2.95	4.25	14.98	3.06
Butanoic acid	0.76	n.d	0.89	n.d	n.d	0.48	0.21	0.61	1.22	n.d
Decanoic acid	0.94	0.44	0.83	0.52	0.43	0.72	0.31	0.40	0.37	0.62
Formic acid	0.97	5.52	0.50	0.98	n.d	4.95	0.54	0.73	3.52	n.d
Heptanoic acid	n.d	0.23	0.36	0.55	n.d	n.d	0.24	n.d	n.d	0.32
Octanoic acid	2.01	1.49	1.97	2.56	2.54	1.38	2.02	2.00	2.02	2.76
Hexanoic acid	1.89	4.65	10.05	3.14	12.06	5.06	9.97	13.00	22.64	13.95
Nonanoic acid	6.22	3.39	4.50	4.77	3.97	5.70	2.86	3.44	3.39	4.59
**Total Acids**	**20.20**	**27.40**	**28.78**	**19.68**	**23.63**	**39.53**	**19.73**	**26.51**	**52.43**	**26.25**
**Aldehydes**										
2.4-dimethyl-benzaldehyde	n.d	n.d	n.d	15.34	n.d	n.d	n.d	n.d	1.63	3.74
2-hexenal	1.96	1.89	2.84	7.46	8.29	3.53	2.76	2.28	6.58	3.30
Benzaldehyde	n.d	n.d	n.d	0.63	0.76	n.d	n.d	n.d	0.84	0.39
**Total Aldehydes**	**1.96**	**1.89**	**2.84**	**23.43**	**9.05**	**3.53**	**2.76**	**2.28**	**9.05**	**7.43**
**Terpens**										
beta-bisabolene	0.33	0.38	0.40	n.d	n.d	2.61	n.d	0.34	n.d	n.d
**Total Terpens**	**0.33**	**0.38**	**0.40**	**0.00**	**0.00**	**2.61**	**0.00**	**0.34**	**0.00**	**0.00**
**Total Other Compounds**	**12.43**	**35.25**	**1.47**	**13.44**	**0.00**	**9.28**	**0.74**	**3.01**	**0.54**	**0.83**

n.d. not detected; n.a. not available.

## Data Availability

All new research data were presented in this contribution.
